# Securing maize reproductive success under drought stress by harnessing CO_2_ fertilization for greater productivity

**DOI:** 10.3389/fpls.2023.1221095

**Published:** 2023-10-04

**Authors:** Yangyang Li, Pengpeng Zhang, Wenjing Sheng, Zixiang Zhang, Ray J. Rose, Youhong Song

**Affiliations:** ^1^ College of Agronomy, Anhui Agricultural University, Hefei, Anhui, China; ^2^ School of Environmental and Life Sciences, The University of Newcastle, Newcastle, NSW, Australia; ^3^ Centre for Crop Science, Queensland Alliance for Agriculture and Food Innovation, The University of Queensland, Brisbane, QLD, Australia

**Keywords:** *Zea mays L.*, reproductive success, drought stress, CO_2_ fertilization, assimilate allocation, leaf photosynthesis

## Abstract

Securing maize grain yield is crucial to meet food and energy needs for the future growing population, especially under frequent drought events and elevated CO_2_ (eCO_2_) due to climate change. To maximize the kernel setting rate under drought stress is a key strategy in battling against the negative impacts. Firstly, we summarize the major limitations to leaf source and kernel sink in maize under drought stress, and identified that loss in grain yield is mainly attributed to reduced kernel set. Reproductive drought tolerance can be realized by collective contribution with a greater assimilate import into ear, more available sugars for ovary and silk use, and higher capacity to remobilize assimilate reserve. As such, utilization of CO_2_ fertilization by improved photosynthesis and greater reserve remobilization is a key strategy for coping with drought stress under climate change condition. We propose that optimizing planting methods and mining natural genetic variation still need to be done continuously, meanwhile, by virtue of advanced genetic engineering and plant phenomics tools, the breeding program of higher photosynthetic efficiency maize varieties adapted to eCO_2_ can be accelerated. Consequently, stabilizing maize production under drought stress can be achieved by securing reproductive success by harnessing CO_2_ fertilization.

## Introduction

Maize (*Zea mays* L.) is one of the most important cereal crops worldwide, serving as a major source of food, feed, and biofuel, with a total production of 1.16 billion tons from 201.98 million hectares cultivated (FAOSTAT, 2021). Maize production is predominantly influenced by climatic conditions during the growing season, with drought stress having a significant impact on grain yield, comparable to the cumulative effects of all other environmental factors (Boyer and Westgate, 2004). In past climate, maize plants have suffered from drought stress during the individual or multiple growth stages, grain yield losses are most pronounced when drought stress occurs during early reproductive stage (Saini and Westgate, 1999; Kadam et al., 2014; Messina et al., 2019). That can lead to several reproductive development failure irreversibly even though the parent remains alive, especially ovary abortion in maize (Boyer and Westgate, 2004; Shen et al., 2020; Sinha et al., 2021). Yield losses from drought stress at early reproductive stage are foreseen to be as much as 30% based on modelling studies (Lobell et al., 2014). Consequently, securing reproductive success in maize under drought stress is essential for increasing stability of food system.

During the early reproductive stage, drought stress reduces grain numbers in maize are often ascribed to a lack of egg fertilization, resulting in undeveloped ovules (Boyer and Westgate, 2004). Due to pollen water potential always remains lower than parent or silk, female florets show more sensitive to drought stress than male florets, suggesting that the abortion is controlled by female inflorescence under drought stress (Westgate and Boyer, 1986a; Westgate and Boyer, 1986b). A study on the drought tolerance of 18 maize hybrids released during the 1953-2001 period (Campos et al., 2006) showed that genetic yield gains are associated with increased kernels per ear and reduced anthesis–silking interval (ASI) under drought stress at flowering stage. Later in early filling stage, drought stress also reduces kernel number due to less available carbon supply (Boyer and Westgate, 2004; Cakir, 2004). There is abundant evidence that drought stress inhibits photosynthesis (Tang et al., 2023), impairs carbon metabolism (Zinselmeier et al., 1995b; Muller et al., 2011; Shen et al., 2020), and ultimately triggers ovary abortion due to sugar starvation (Boyle et al., 1991; Zinselmeier et al., 1995a; Tang et al., 2023).

With climate change, drought stress is projected to become more frequent, longer, and more severe, posing a huge challenge to sustainable maize production (Harrison et al., 2014; Lobell et al., 2014; Yuan et al., 2023). According to the latest AR6 Synthesis Report, ambient CO_2_ concentration has increased from the preindustrial level of 280 to 410 ppm today (IPCC, 2023), and is considered a major driving force of drought stress (Kadam et al., 2014; Yuan et al., 2023). However, a recent meta-analysis by Ainsworth and Long (2021) has found that elevated CO_2_ (eCO_2_) can enhance the productive capacity of C4 crops under drought stress, as eCO_2_ significantly improves water use efficiency (WUE), based on over 250 observations from free-air CO_2_ enrichment (FACE) experiments worldwide. Consequently, rising CO_2_ concentration provides a unique opportunity to maintain maize productivity under drought stress (Leakey et al., 2004; Lobell et al., 2014; Gonsamo et al., 2021). Given the limitations of FACE experiments, far less is known about how maize plants achieve their reproductive success under the interactive effects of eCO_2_ and drought stress, and what the contribution of CO_2_ fertilization? In this review, we summarize studies that have explored the photosynthetic production capacity and reproductive development in maize under drought stress, and propose strategies to secure reproductive success under drought stress in maize by harnessing CO_2_ fertilization for greater productivity.

## Effects of drought stress during reproductive stage on leaf photosynthesis and grain yield in maize

The reproductive stage is critical for maize as it determines kernel setting and final yield potential. Drought stress during this stage can cause a significant reduction in both photosynthesis and grain yield in maize, the extent to which depends on the severity and duration of the stress period (**Table 1**). It is evident that reproductive stage of maize is more sensitive to drought than vegetative stage and the concurrent decrease in photosynthesis and yield under drought stress occurs consistently in maize.

**Table 1:** The decline in photosynthesis and yield for maize under drought stress.

Stress timing	Stress severity and duration	Photosynthesis	Yield	References
26 d after sowing	10-30% soil moisture content for 10 d	16-100%		(Kakani et al., 2011)
Jointing stage	35-60% soil relative water content for 30 d	12-52%		(Tang et al., 2023)
Anthesis stage	<50% plant available soil water content for 30 d, 50% field capacity for 15 d, –0.51 MPa soil water potential until final fertilization	16-43%		(Manderscheid et al., 2014), (Hussain et al., 2019), (Liu et al., 2022)
Filling stage	55-75% moisture content until maturity	2%-25%		(Ye et al., 2020)
Vegetative stage	no irrigation at vegetative stage, 85% relative turgidity for 4 days		9-17%	(Cakir, 2004), (Claasen and Shaw, 1970)
Jointing stage	35-60% soil relative water content for 30 d, no irrigation for 48 d, no irrigation until harvesting		46-99%	(Tang et al., 2023), (Monneveux et al., 2006), (Kamara et al., 2003)
Before anthesis	no irrigation for 14 d, no irrigation at anthesis stage		50-75%	(Castiglioni et al., 2008), (Cairns et al., 2013)
Anthesis stage	no irrigation at anthesis stage, 60% plant available soil water for 26 d, <50% plant available soil water content for 30 d, 40% irrigation (0.06 m^3^ m^-3^ VWC) for 13 d		36-65%	(Cakir, 2004), (Campos et al., 2006), (Manderscheid et al., 2014), (Bheemanahalli et al., 2022)
Silking stage	85% relative turgidity for 4 d, no irrigation at silking stage		43-53%	(Claasen and Shaw, 1970), (Ghooshchi et al., 2008)
Filling stage	85% relative turgidity for 4 d, no irrigation at filling stage, 60% plant available soil water for 26 d		22-39%	(Claasen and Shaw, 1970), (Cakir, 2004), (Campos et al., 2006)

### Photosynthesis under drought stress

The response of maize photosynthesis to drought stress during the reproductive period is more intense than during the vegetative period, as the drought recovery capacity of leaf photosynthesis after the tasseling stage is relatively poor (Cai et al., 2020). Moreover, the influence on photosynthesis is more profound at the tasseling stage than at the jointing and milk stages, even at the same drought level (Myers et al., 2017). These results suggest that drought stress during reproductive stage, particularly at the flowering stage, threatens kernel setting by limiting leaf photosynthesis and thus carbohydrate supply (**Table 1**). This limitation results from a decrease in leaf expansion, impaired photosynthetic machinery, premature leaf senescence, and a related decrease in assimilate production (Cai et al., 2020).

**Figure 1:**
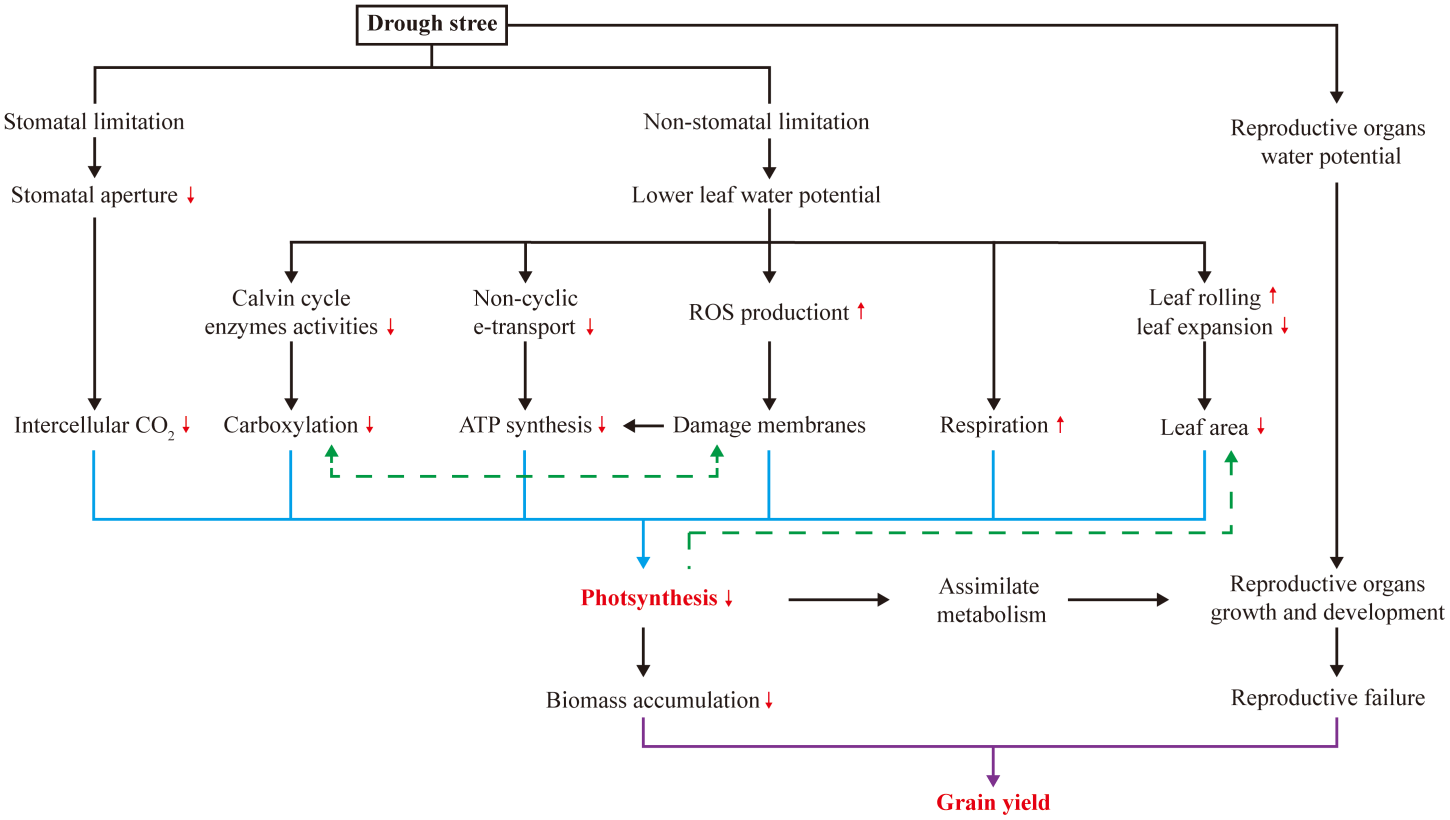
Photosynthesis and grain yield of maize under drought stress.

Both stomatal and non-stomatal limitations on leaf photosynthesis occur under drought stress (**Figure 1**), and photosynthesis is prone to non-stomatal limitations to photosynthesis in maize (Farooq et al., 2009; Lopes et al., 2011; Song et al., 2020b). This suggests that processes other than CO_2_ uptake are being affected. Drought stress typically limits CO_2_ uptake by inducing stomatal closure in leaves (Muller et al., 2011), but the ability of maize to concentrate CO_2_ around Rubisco in the bundle sheath cells mitigates this effect (Leakey et al., 2019). Regarding non-stomatal limitation, drought stress causes changes in photosynthetic pigments and components (Manderscheid et al., 2014; Ye et al., 2020; Bheemanahalli et al., 2022), damages photosynthetic apparatus (Ye et al., 2020), diminishes activities of Calvin cycle enzymes (Kakani et al., 2011; Cai et al., 2020; Correia et al., 2021), and induces photorespiration (Sicher and Barnaby, 2012), all of which contribute to reduced photosynthesis. In addition, to survive under drought stress, maize expends a considerable amount of energy to cope with it through respiration (Farooq et al., 2009). Another critical effect of drought stress is the imbalance between reactive oxygen species (ROS) production and antioxidant defense (Sicher and Barnaby, 2012; Ye et al., 2020; Sinha et al., 2021), which results in ROS accumulation and induces oxidative stress in proteins, membrane lipids and other cellular components. Variation in these non-stomatal limitations affects photosynthesis and assimilate accumulation, providing potential targets for increasing maize yield under drought stress (**Figure 1**).

### Grain yield under drought stress

Many yield-determining physiological processes in plants respond to drought stress (**Figure 1**). In maize, early reproductive stage is highly susceptible to drought stress, resulting in a pronounced loss of kernel number and, consequently, grain yield (Saini and Westgate, 1999; Boyer and Westgate, 2004; Setter et al., 2011). As presented in **Table 1**, drought stress occurring during the rapid vegetative growth period causes a little loss of final grain yield by 9-17%, while more significant losses during the filling stage by 22-39% are mostly through reducing kernel size, and severe losses during early reproductive stage by 36-99% result from reductions in kernel numbers. Large agricultural losses can occur during the whole reproductive stage, but the irreversibility of the early events is particularly damaging. When drought stress occurs during the early reproductive stage, losses in kernel number are attributed to long anthesis-silking interval (ASI) (Boyer and Westgate, 2004; Duvick, 2005; Fuad-Hassan et al., 2008) and disruption of ovarian carbon metabolism (Muller et al., 2011; Liu et al., 2022); while during filling stage, grain yield losses are attributed to the reduced kernel weight that limited by insufficient assimilate supply (Boyer and Westgate, 2004). Consequently, reproductive success in maize can be mitigated by increasing photo-assimilation supply (**Figure 1**).

## Reproductive drought tolerance mechanisms in maize

In past decades, breeding has led to the development of maize genotypes with increased drought tolerance, primarily attributable to enhanced reproductive resilience (Zinselmeier et al., 1995b; Lobell et al., 2014; Messina et al., 2019; Sinha et al., 2021). For example, conventional breeding has produced hybrid lines less susceptible to drought stress, with the improvements primarily resulting from increased assimilate accumulation in reproductive organs and rapidly silking (Bänziger et al., 2002; Cattivelli et al., 2008; Araus et al., 2012; Nuccio et al., 2015). Consequently, constitutive drought tolerance mechanisms may exist in maize and are closely related to the establishment of reproductive structures (Bänziger et al., 2002). Under drought stress, reproductive organ expansion being affected earlier and more intensively than photosynthesis and metabolism (Fuad-Hassan et al., 2008; Muller et al., 2011), resulting in reproductive failure, i.e., failure to pollinate or post-pollination ovary abortion (Zinselmeier et al., 1995b; Boyer and Westgate, 2004; Fuad-Hassan et al., 2008; Oury et al., 2016; Turc et al., 2016; Yang et al., 2018; Bheemanahalli et al., 2022). Therefore, securing ovary and silking success poses a critical importance for enhancing reproductive drought tolerance in maize under drought stress.

### Greater assimilate import into ear

**Figure 2:**
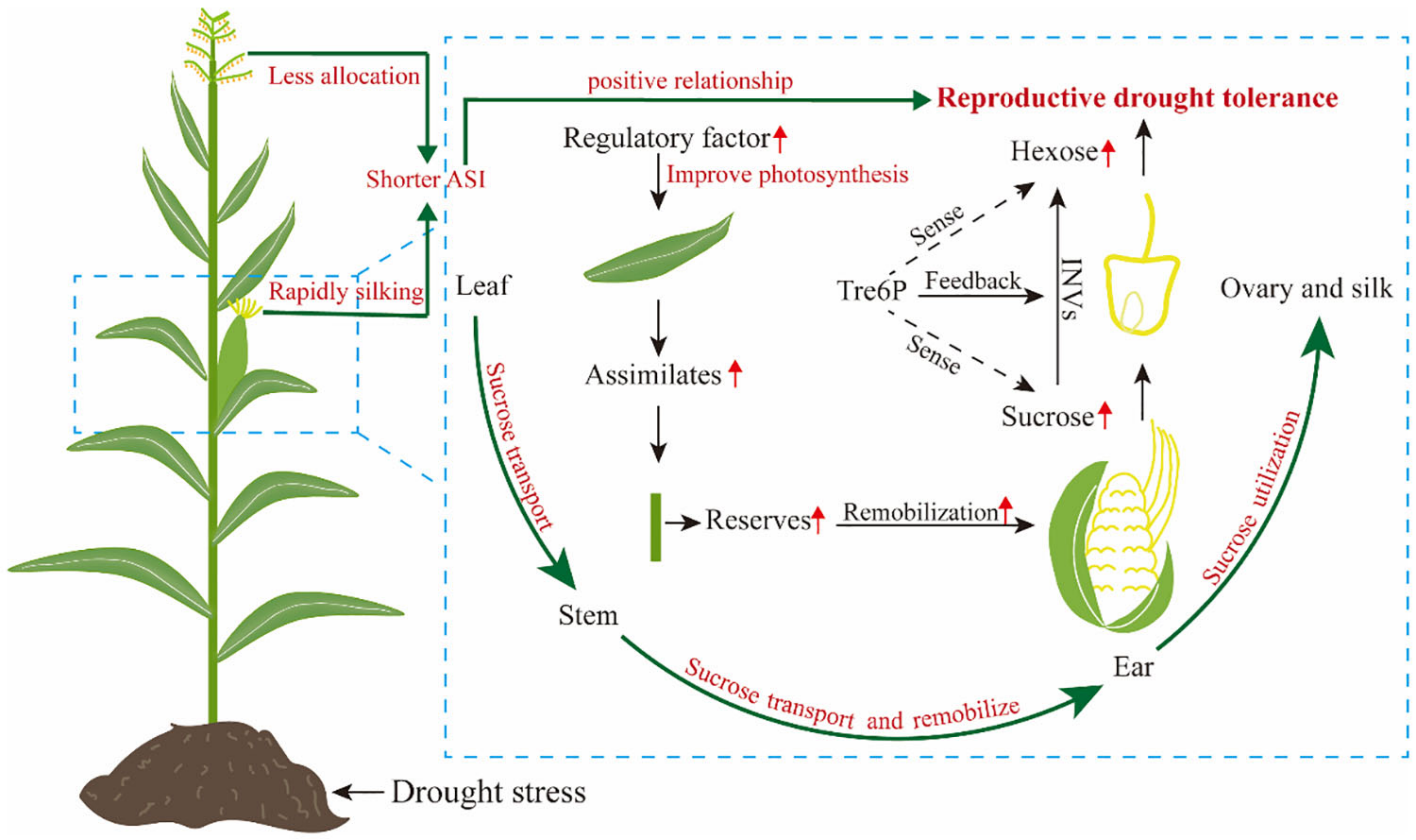
Diagram of reproductive drought tolerance mechanism of maize.

Assimilate partitioning and transportation under drought stress contribute significantly to reproductive growth and development of maize, particularly when an inadequate assimilate supply to ear causes severe grain yield losses (Mclaughlin and Boyer, 2004b; Boyer and Mclaughlin, 2007; Sinha et al., 2021). Multiple lines of evidence suggest that maintaining ear development under drought stress and achieving high seed set are related to maintenance of sugar supply (Zinselmeier et al., 1995a; Boyer and Mclaughlin, 2007). For example, a recent study by Tang et al. (2023) demonstrated that varying degrees of drought stress decreased carbohydrate supply in developing ear by 12%-63%, resulting in ovary abortion, particularly apparent on the tip of maize ear. In addition, two prominent studies have shown that overexpression of trehalose-6-phosphate phosphatase in developing maize ear using a floral promoter decreased trehalostrehalose-6-phosphatephosphate, leading to increased sucrose concentration by regulating assimilate partitioning, and the engineered trait improved yields from 31% to 123% under drought stress (Nuccio et al., 2015; Oszvald et al., 2018). Taken together, reproductive success is closely related to the assimilate flux to the young ear around flowering under drought stress, and concurrent photosynthesis is required to maintain this flux (Bänziger et al., 2002). In maize, overexpression of ZmNF-YB16 (Wang et al., 2018) and Nicotiana protein kinase (NPK1) (Shou et al., 2004) can improve drought tolerance and yield by enhancing photosynthesis (**Figure 2**).

However, the relationship between sugar content and reproductive growth is intricate, and ovary abortion is not solely result from limitations in sugar supply (Lopes et al., 2011; Muller et al., 2011). Ovary abortion can also occur due to disturbed sugar-to-starch synthesis, even when there is an adequate sucrose supply (Zinselmeier et al., 1999). The reason is that factors i.e. phytohormones and plant metabolites other than an inadequate supply of assimilates also initiate the ovary abortion process (Feng et al., 2011a; Shabbaj et al., 2022). For example, the addition of 1-aminocyclopropane-1-carboxylic acid to the culture medium results in the evolution of ethylene *in vitro*, which causes abortion and reduces mature kernel mass in maize (Cheng and Lur, 1996). Amino acids and their derived metabolism e.g. polyamine is also found that play a crucial role in the regulation of early endosperm development under eCO_2_ (Liang and Lur, 2002; Geng et al., 2020). The ovary abortion caused by phytohormones and plant metabolites largely due to a complex relationship with sugar stream and availability (Feng et al., 2011a). It is clear that increasing assimilate supply by photosynthesis to ear will increase maize reproductive drought tolerance, while maintaining a steady sugar stream and its use in maize reproductive tissues are equally important for reproductive drought tolerance (**Figure 2**).

### More available sugars for ovary and silk use

Sugar availability in the developing ovary and silk have been correlated with the incidence of ovary abortion under drought stress (Andersen et al., 2002; Feng et al., 2011b; Kakumanu et al., 2012; Oury et al., 2016). In this process, invertases (INVs) serve as an important part of hexose supply that maintains growth and developmental processes for ovary and silk (Andersen et al., 2002; Oury et al., 2016). Generally, drought stress reduces INVs activity in the ovaries, leading to a decrease in hexose, depletion of starch reserves in ovary, and abortion of ovaries (Boyer and Mclaughlin, 2007; Sinha et al., 2021). Not just that, the decreased invertase activity accounts for the slower sucrose uptake in ovary under drought stress (Boyer and Westgate, 2004). A related work showed that increased INVs in drought-stressed maize can maintain reproductive organ hexose concentration at an “unstressed” level, contributing to enhance reproductive drought tolerance (Kakumanu et al., 2012). Specifically, the ivr2 gene, which encodes an acid-soluble invertase, is verified to be functional in enhancing maize ovary drought tolerance (Qin et al., 2004; Li et al., 2011). Additionally, the decreased sugar availability can delay silk growth under drought stress, leading to an increased ASI and ultimately resulting in ovary abortion (Muller et al., 2011; Oury et al., 2016; Messina et al., 2019). It follows that the differences of sugar utilization in ovary and silk determine the “live or die” fate of maize ovary siblings during sugar competition under drought stress (Shen et al., 2020; Shen et al., 2023).

In addition to sucrose availability itself, the signaling of sucrose availability is critically important for ovary development under drought stress (Andersen et al., 2002; Bledsoe et al., 2017). Trehalose-6-phosphate (Tre6P) plays a central role in sugar sensing, leading to the proposal that Tre6P acts a signal of sucrose availability, and in turn regulates sucrose production and utilization (Bledsoe et al., 2017). In maize, several works showed that expression of Tre6P phosphatase in ovary improves its drought tolerance (Nuccio et al., 2015; Bledsoe et al., 2017; Oszvald et al., 2018). Overall, sugar utilization in ovary is as important as the continuous sugar supply for reproductive drought tolerance (**Figure 2**).

### Higher capacity to remobilize assimilate reserve

Drought stress causes maize plants scarcely any assimilate accumulated, but kernels can continue to fill for some time results from the assimilate reserves (Mcpherson and Boyer, 1977). A further study by Sinclair et al. (1990) found kernel yield is closely related to assimilate accumulation in stem under drought stress. Therefore, assimilate reserves in maize are especially critical for drought tolerance. Although stem serves as a major sink for assimilate during the vegetative phase of maize growth, assimilate stored in the stem can be remobilized to reproductive organs (Ribaut et al., 2009). This remobilization and its contribution to final kernel yield are significantly affected by drought stress (Mclaughlin and Boyer, 2004a; Boyer and Mclaughlin, 2007; Ribaut et al., 2009; Golzardi et al., 2017). Inhibition of pre-fertilization ear growth due to drought can be in partially overcome by sugar supply via the stem, which is transported to the ear (Mclaughlin and Boyer, 2004a; Boyer and Mclaughlin, 2007). Early seminal studies by Boyle et al. (1991) and Zinselmeier et al. (1995a) suggested that re-establishing the sugar stream via the stem can prevent ovary abortion, and seed set is largely preserved in maize. Despite remobilization of stem assimilates to ear is possible in maize, remobilization efficiency still requires further investigation. As demonstrated in sorghum (Borrell et al., 2006), an increase in stem diameter may favor assimilate reserve and remobilization to developing grains under drought stress. Consequently, maize genotypes with a better ability to store and remobilize assimilates in stem will exhibit greater drought stress tolerance (**Figure 2**).

## Securing reproductive success by harnessing CO_2_ fertilization

As summarized above, maize reproductive success depends on three aspects: (i) greater assimilate import into ear; (ii) more available sugars for ovary and silk use; (iii) higher capacity to remobilize assimilate reserve. Several CO_2_ enrichment studies with maize conducted under controlled environment conditions have demonstrated an increase in photosynthesis and grain yield under drought stress (Leakey et al., 2004; Markelz et al., 2011; Manderscheid et al., 2014), but no significant effect was observed when the plant was not experiencing drought stress (Leakey et al., 2006; Markelz et al., 2011). As a result, a strong correlation exists between reproductive success under drought stress and CO_2_ fertilization.

### Boosting leaf photosynthesis by harnessing CO_2_ fertilization under drought stress

Drought stress can inhibit maize photosynthesis by limiting the water availability and inducing a set of related limitations. FACE experiments with drought stress have indicated that eCO_2_ can indirectly enhance maize photosynthesis through improving plant water relations, thus mitigating the negative effects of water scarcity on growth and photosynthetic system, (Kimball et al., 2002; Leakey et al., 2006; Manderscheid et al., 2014; Ainsworth and Long, 2021). WUE is a constraint and target for improving crop resilience and productivity (Leakey et al., 2019). In maize, photosynthesis is saturated at the current ambient CO_2_ level due to its CO_2_ concentrating mechanism, so there is no direct stimulation of photosynthetic carbon gain and yield (Manderscheid et al., 2014). However, eCO_2_ decreases maize stomatal conductance (g_s_) by 22%, mostly through reduced aperture (Ainsworth and Rogers, 2007), resulting in a 16-68% decrease in transpiration (Kimball and Idso, 1983), greatly improving WUE and conserving soil moisture (Leakey et al., 2006; Leakey, 2009). This indicates that the potential benefits of greater WUE associated with lower g_s_ and equivalent photosynthesis can be realized in maize; such mechanisms may counteract the development of drought stress under eCO_2_ and prevented the inhibition of photosynthesis observed under ambient CO_2_.

**Figure 3:**
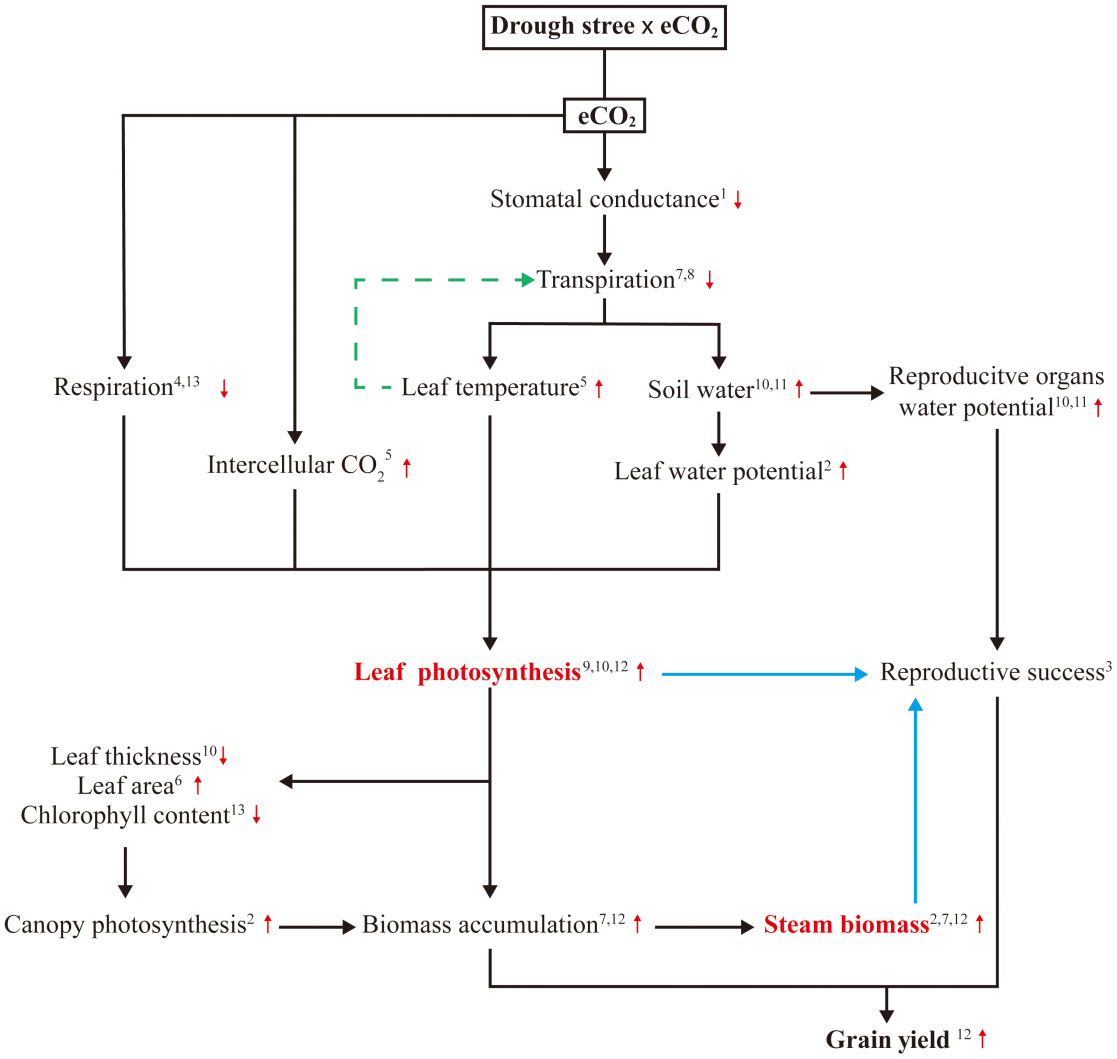
Summary of the main mechanisms contributing to reproductive success in maize exposed to eCO_2_ under drought stress. ^1^(Ainsworth and Rogers, 2007); ^2^(Allen et al., 2011); ^3^(Boyer and Westgate, 2004); ^4^(Cousins et al., 2001); ^5^(Ghannoum et al., 2000); ^6^(Kadam et al., 2014); ^7^(Kim et al., 2006); ^8^(Kimball and Idso, 1983); ^9^(Kimball et al., 2002); ^10^(Leakey et al., 2006); ^11^(Leakey, 2009); ^12^(Manderscheid et al., 2014); ^13^(Sun et al., 2022).

There is also evidence that eCO_2_ directly stimulate maize leaf photosynthesis by reducing the time needed for stomatal opening under dynamic irradiance (Leakey et al., 2005), decreasing stomatal aperture (Ainsworth and Rogers, 2007), increasing intercellular CO_2_ concentration (C_i_) and leaf temperature, and altering diurnal CO_2_ fixation patterns (Ghannoum et al., 2000), resulting in increased photosynthesis. Decreasing respiration has been a target for improving photosynthesis (Joshi et al., 2023), early studies attributed eCO_2_ enhanced photosynthesis of immature fully exposed leaves to suppressed photorespiration and enhanced energy use efficiency from decreased leakage of CO_2_ from bundle sheath cells and reduced over-cycling of the C4 pump (Cousins et al., 2001; Ainsworth and Rogers, 2007). A more recent study showed that eCO_2_ led to an 8.4% reduction in day respiration rate and a 16.2% reduction in dark respiration, as decreased leaf N and chlorophyll contents (Sun et al., 2022). In addition to these physiological traits, eCO_2_ promotes leaf area (Kadam et al., 2014) and decreases leaf thickness (Leakey et al., 2006), combining with low leaf chlorophyll concentration, thereby providing more surface area for light interception and allowing more light penetration to lower layers of a dense canopy, ultimately resulting in higher maize canopy photosynthesis and biomass accumulation (Kim et al., 2006; Allen et al., 2011). eCO_2_ maintains water balance by increasing the accumulation of compatible solutes including glucose, fructose, β-alanine, and γ-aminobutyric acid (GABA) in addition to enhancing photosynthetic properties, thereby improving drought tolerance (Abdelhakim et al., 2022). Consequently, the increase in photosynthesis under eCO_2_ and drought stress involves a complex process with multiple mechanisms (**Figure 3**). Although not yet fully understood, these mechanisms offer promising avenues for providing adequate supply of assimilate by enhancing leaf photosynthesis under drought stress and mitigating the negative effects of water scarcity on reproductive growth and productivity.

The impact of eCO_2_ on maize productivity was commonly positive when soil water content be limiting for growth and evaporative demand under ambient CO_2_. The response of maize to eCO_2_ is therefore likely to vary among different periods. For example, under limited water conditions, in the experiments by enriching CO_2_ since emergence, eCO_2_ increased maize canopy daily photosynthesis by 9% and reduced canopy transpiration by 22% on 26 days after emergence (DAE) (Allen et al., 2011), and caused an enhancement of leaf photosynthesis by 21% on 37 DAE and 11% on 48 DAE (Leakey et al., 2004). When CO_2_ enrichment occurred from 23 DAE to harvest, eCO_2_ significantly increased final biomass by 24% and grain yield by 41% (Manderscheid et al., 2014). However, eCO_2_ may negatively affect maize yields when eCO_2_ partially offsets the yield gaps caused climate extremes (drought, heat) due to excessively high CO_2_ concentrations. A simulation study demonstrated that severe drought pattern caused by eCO_2_ (550 ppm) accelerated early maturity and had the greatest impacts on maize yield (Harrison et al., 2014). Under eCO_2_ (845 ppm) scenario, high temperature and heat stress replace the dominant stress of drought on maize, resulting in prominent yield losses (Jin et al., 2017). These results highlight the importance of considering interaction of eCO_2_ with other environmental variables, especially extreme events, in future agronomic adaptation and mitigation strategies.

### Increasing and remodeling assimilate reserves in stem

When drought stress occurs during reproductive stage in maize, having the adequate assimilate reserves in stem appears to be particularly important, as reproductive cellular activities and respiration continue to demand substrates (Boyer and Mclaughlin, 2007; Farooq et al., 2009). Assimilate accumulation of maize stem under different drought stress conditions has been shown to decrease by 16-24% (Kamara et al., 2003), 16-44% (Ge et al., 2011) and 12-63% (Tang et al., 2023) due to decreased photosynthesis capacity, and the corresponding yield decreased by 49-99%, 20.4-84.5%, and 46-99%, respectively. Can this reduction be reversed? As previously mentioned, eCO_2_ can increase assimilate supply in maize by enhancing photosynthesis under drought stress. Based on CO_2_ enrichment experiments, significant increases in biomass accumulation in maize stem under drought stress have been reported, range from 9% (Manderscheid et al., 2014) to 20% (Allen et al., 2011). These results imply that eCO_2_ allows maize to produce and store more assimilate in stem before flowering stage under drought stress. When drought stress occurred during the reproductive period, stress can stimulate the remobilization of pre-stored assimilate reserves (Yang et al., 2001), subsequently, large amounts of carbohydrate were moved from the stems to the grain, compensating for the lack of current photosynthesis (Boyer and Westgate, 2004). These evidences suggest that eCO_2_ improves the potential for remobilization of assimilate reserves in maize stem to support kernel growth under drought stress, providing a promising strategy for enhancing maize productivity and food security under drought stress (**Figure 3**).

### The effect of eCO_2_ on grain quality

Although eCO_2_ increases photosynthesis and carbohydrate content, but not mineral elements, thus alters the stoichiometric balance of nutrients in crop grains and has a significant impact on human nutrition (Loladze, 2002). Gradually, the effect of eCO_2_ on crop quality is becoming a hot topic of research (Myers et al., 2017; Zhu et al., 2018). Most studies are in favour of the idea that eCO_2_ leads to an increase in carbohydrate content, which dilutes mineral and protein concentrations in plant tissues (Taub et al., 2008). For example, eCO_2_ decreased the mineral concentrations such as nitrogen and phosphorus in maize grains (Abebe et al., 2016), iron and zinc in rice grains (Zhu et al., 2018), and magnesium, copper, calcium, and manganese in wheat grains (Guo et al., 2021). Protein content decreased in wheat and rice, but not significantly in soybean and pea, indicating the ability of legumes to convert excess carbon for nitrogen fixation (Myers et al., 2014). In maize, eCO_2_ did not significantly affect the protein content of maize grains, but increased the oil content (Qiao et al., 2019). Interestingly, a recent study found that the negative effects of eCO_2_ on grain quality in rice and wheat grains were compensated by the positive effects of elevated temperature (Guo et al., 2021). Limited by the FACE facilities, there remains substantial uncertainty about the interacting consequences of eCO_2_, multiple environmental factors, and cropping practices on crop quality. More comprehensive replicated experiments are needed to clarify the mechanisms and environmental conditions that lead to lower nutrient levels in eCO_2_.

## Adapting to rising CO_2_ concentration

Rising CO_2_ concentration has the potential to boost photosynthesis and assimilate accumulation, and thus secure reproductive success and productivity in maize under drought stress as elaborated above. However, environmental factors and leaf photosynthetic capacity determine the extent to which photosynthesis responds to eCO_2_. To capitalize on this potential, optimizing planting methods and selecting maize varieties that can adapt to rising CO_2_ concentration hold the same importance. In the following sections, we will explore strategies for achieving such adaptation, enabling to harness the enhanced productivity from rising CO_2_ concentration while mitigating the various detrimental impacts of climate change on global food supply.

### Optimizing planting methods

Optimizing cropping systems to adapt to an elevated CO_2_ environment involves integrating the implications of the various technological possibilities associated with the system components and identifying and exploiting their interactions at the population scale (Hammer et al., 2021). Water availability directly restricts maize growth and development. Fortunately, conservation agriculture (e.g., minimum tillage (Lobell et al., 2014), mulching (Niu et al., 2020), and cover cropping (Manderscheid et al., 2014)) and precision irrigation techniques (e.g., surface drip irrigation (Liu et al., 2023), shallow‐buried drip irrigation (Ayars et al., 2015), alternate furrow irrigation (Golzardi et al., 2017), and micro-sprinkling irrigation (Li et al., 2021a)) have been shown to help retain soil moisture, reduce evaporation, and improve water use efficiency. In a future eCO_2_ scenario, these water-saving measures can allocate limited water supplies to irrigate larger area of maize, potentially increasing overall maize yields in water-limited areas. Given the large amount of nitrogen invested by plants in Rubisco and Rubisco’s role as a C fixing enzyme (Evans and Clarke, 2019), it is not surprising that the balance between photosynthetic utilization and nitrogen status plays an important role in shaping the plant’s response to eCO_2_. Ainsworth and Long (2005) reported that the stimulation of light-saturated CO_2_ uptake (A_sat_) and maximum carboxylation rate (V_cmax_) at eCO_2_ was 23% and 85% lower in plants grown with a low nitrogen supply, respectively. Furthermore, Markelz et al. (2011) pointed out that drought damage to maize photosynthesis is exacerbated by nitrogen limitation and improved by eCO_2_. Nitrogen limitation can cause carbon sink limitation at the individual plant level, thereby reducing the actual growth achieved by eCO_2_ (Ainsworth and Rogers, 2007). Matching the increased C supply with additional nitrogen at eCO_2_ is key to avoiding sink limitation (Leakey et al., 2009). It follows that adequate nitrogen supply is an effective measure for maize to adapt to eCO_2_. While these planting methods and agricultural practices can help adapt maize production to eCO_2_ and drought stress, they still need to be further tested in relation to local conditions and specific crop requirements.

### Mining natural genetic variation

Genetic variation in crops responses to eCO_2_ is crucial for future breeding efforts aimed at improving productivity (Ainsworth and Long, 2021). Under eCO_2_ environment, notable variation in grain yield has been observed, ranging from 46% to 127% for three maize cultivars (Vanaja et al., 2015), 3% to 36% for eight rice cultivars (Hasegawa et al., 2013) and from 0% to 24% for nine soybean genotypes (Bishop et al., 2015). Both studies demonstrated a similar dependence of yield response to eCO_2_ on sink capacity, indicating that sink capacity in these seed crops is a key limitation to yield responsiveness to eCO_2_ in the field. However, the mechanisms driving greater yields at eCO_2_ in wheat differ from those in soybean and rice. A study by Tausz-Posch et al. (2015) found that eCO_2_ stimulated grain yield increase in a freely tillering cultivar exclusively due to an increase in fertile tiller number, while yield stimulation in a restricted tillering cultivar is additionally associated with increased kernel weight and kernel numbers per spike. In addition, a greater performance response to eCO_2_ is observed in wheat cultivars selected for higher transpiration efficiency (Tausz-Posch et al., 2012). As a result, more comprehensive screening of the vast genetic variation in essential crops will likely reveal significant differences in CO_2_ response that can be utilized in breeding programs. Attaining the theoretical yield response offered by rising CO_2_ levels may be vital for addressing the anticipated supply-demand gap as the century unfolds (Ray et al., 2013).

### Utilizing genetic engineering approach

Though the sufficient natural genetic variability in crops can be used in breeding to increase sink strength to counteract the feedbacks from increased photosynthetic potential under eCO_2_, there is limited time for conventional breeding to adjust to rapidly rising CO_2_ concentration (Ainsworth and Rogers, 2007; Ainsworth and Long, 2021). Advanced genetic engineering tools may be necessary to design and implement new photosynthetic system for better efficiency under eCO_2_ (Long et al., 2015; Zhu et al., 2022). Rubisco catalyzes ribulose bisphosphate (RuBP) carboxylation and oxygenation, representing an evolutionary preferred choice in optimizing photosynthesis (Long et al., 2015; Zhu et al., 2022). Under eCO_2_, leaf Rubisco content decreases by 20% due to reduced leaf nitrogen content (Ainsworth et al., 2002). A recent transgenic study by Yoon et al. (2020) found that upregulation of Rubisco content in rice causes increased photosynthesis and yield, suggesting that advanced genetic engineering technology has potential to overcome the reduced Rubisco content under eCO_2_. In scenario with simultaneous increases in CO_2_ and temperature, the limitation of CO_2_ assimilation has a tendency to RuBP regeneration instead of Rubisco (Perdomo et al., 2017; Ainsworth and Long, 2021). Sedoheptulose-1,7-bisphosphatase (SBPase) has been reported to be related to RuBP regeneration (Long et al., 2015) can thus be a target for manipulation to increase assimilation without additional resources. Transgenic upregulation of SBPase in soybean, allowing to enhance photosynthesis, thereby protecting against temperature-induced yield loss under eCO_2_ (Köhler et al., 2017). In addition, upregulation of the Rieske Fe-S protein of electron transport (Simkin et al., 2017) and the H-protein of the glycine cleavage system (López-Calcagno et al., 2019) can also increase RuBP regeneration rates. Taken together, these results highlight genetic engineering can efficiently modify key targets to further maximizing photosynthesis under eCO_2_.

### Combining with plant phenomics

To fully exploit the potential of genetic engineering tools, greater emphasis should be placed on applying appropriate secondary traits and high-throughput phenotyping tools to identify germplasm with high photosynthetic capacity under eCO_2_ (Araus et al., 2012; Meacham-Hensold et al., 2020; Zhu et al., 2022; Munne-Bosch and Villadangos, 2023). Complex traits, such as grain yield, drought tolerance, and high photosynthetic efficiency, appear to have low heritability due to significant genotype × environment interactions (Lopes et al., 2011; Leakey et al., 2019). Using secondary traits as the primary phenotypic traits may be a viable alternative for selecting high photosynthetic efficiency. This approach can improve the selection efficiency and precision because the heritability of some secondary traits remains higher than that of complex traits, has exhibits sufficient genetic variability, and is genetically correlated with complex traits (Araus et al., 2012; Munne-Bosch and Villadangos, 2023). Advanced plant phenotyping technologies allow to predict physiological and anatomical traits related to photosynthetic efficiency. For example, typical gas exchange system (Kumagai et al., 2022), emerging multispectral (Fu et al., 2022), hyperspectral (Yendrek et al., 2017; Meacham-Hensold et al., 2020), fluorescence (Li et al., 2020; Xia et al., 2023), and thermal (Munne-Bosch and Villadangos, 2023) sensors.

Based on LI-6800 Portable Photosynthesis System, combining with PACiR (Stinziano et al., 2017) and DAT (Saathoff and Welles, 2021) techniques, measurements of V_cmax_ and maximal linear electron transport rate (J_max_) can be obtained in 5 min, and possibly even faster compared to typical Steady State technique. To make the measurement more convenient, Xia et al. (2023) developed an least-squares SVM (LSSVM) model that can obtain Fv/Fm from chlorophyll a fluorescence signals measured without dark adaptation. Although these methods have greatly improved their efficiency compared to traditional methods, they still do not allow for the rapid measurement of more species or genotypes within a species to enable the study of genetic diversity. Interestingly, at leaf level, Kumagai et al. (2022) constructed a predictive model for V_cmax_ and J_max_ by coupling spectral vegetation indices and machine learning methods. The results showed that hyperspectral reflectance captured the biochemical acclimation of leaf photosynthesis to high temperature in the field. Using a similar method, Yendrek et al. (2017) accurately predicted chlorophyll content, N content, specific leaf area and V_cmax_ of maize leaf, enabling to phenotyping over 1000 rows during midday hours in only 2 to 4 days. The widespread application of PAM fluorescence in quantitative photosynthesis has further stimulated interest in passive detection of chlorophyll fluorescence under solar irradiation (Fu et al., 2022), namely solar-induced fluorescence (SIF). Camino et al. (2019) estimated V_cmax_ for both rainfed and irrigated wheat trials by combining SIF and hyperspectral images through the inversion of the SCOPE model. At plot level, Based on time-synchronized hyperspectral images and irradiance measurements, Fu et al. (2021) purposed an alternative yet promising approach to monitor tobacco photosynthetic capacity (V_cmax_ and J_max_). At canopy level, Li et al. (2020) used solar induced fluorescence (SIF) and hyperspectral imagery to characterize the maize canopy photosynthetic light use efficiency. To detect the effect of drought stress on maize and soybean leaf physiology, Sobejano-Paz et al. (2020) used thermal imaging and machine learning techniques (PLS-R) to assess canopy evapotranspiration, leaf transpiration, stomatal conductance, photosynthesis, chlorophyll content and morphological properties. The results showed that this method can help to parameterize canopy photosynthesis or evapotranspiration models, and identify different photosynthetic processes in response to drought. Despite these techniques can high-throughput phenotyping secondary traits that link leaf photosynthetic capacity to underlying genetics, and thus improve the efficiency of crop photosynthesis improvement in target CO_2_ concentration environment; it is not yet clear whether they have the precision needed to infer small changes in photosynthesis.

### Modelling assists breeding

Exploiting genetic variation in crop yield responses to eCO_2_ necessitates screening diverse germplasm and structured populations to identify the genomic regions associated with greater yield quantity and quality under such conditions (Leakey et al., 2009; Kimball, 2016). Although conducting experiments can be challenging due to the size of individual FACE plots and potential variation between and within them, these obstacles can be overcome by applying a multi-scale modelling approach (Toreti et al., 2020). Nearly 40 years of FACE experiments have generated a vast database and insight into potential mechanisms of plant responses to eCO_2_, which can be invaluable for constructing such models (Leakey et al., 2009; Toreti et al., 2020). Relevant studies have been reported, combining gene network, metabolic and leaf-level models, Kannan et al. (2019) predicted the impacts of *Gm-GATA2* gene regulatory change on soybean photosynthesis under eCO_2_. Furthermore, Song et al. (2020a) used a 3D canopy model to reveal synergistic effects of CO_2_ and light on soybean photosynthesis, found that eCO_2_ improved canopy photosynthesis through increased leaf area index at early developmental stages, while canopy photosynthesis was associated with a higher proportion of leaves in a canopy limited by Rubisco carboxylation at later developmental stages. This suggests modifying Rubisco can further routes for maximizing photosynthesis under eCO_2_. Constructing multi-scale models facilitates the connection between genomics and phenomics (Hammer et al., 2021), and increases the predictability of plant systems (Messina et al., 2018). In addition to eCO_2_ effects, the complex interactions of eCO_2_, temperature, water and nitrogen on crop processes should also be considered in crop models (Toreti et al., 2020). For example, Castaño-Sánchez et al. (2020) used three crop models (CropSyst, DSSAT-M and IFSM) to assess the response of maize yield and evapotranspiration to eCO_2_, and found that models using radiation use efficiency (DSSAT-M, IFSM) and models using transpiration use efficiency (CropSyst) to limit crop growth both overestimated maize growth. However, by coupling photosynthesis, stomatal conductance and transpiration models, Li et al. (2021b) suggested the use of a coupled model predicted rice canopy gas exchange processes under eCO_2_ and warming temperature conditions more accurately than an uncoupled photosynthesis/transpiration model. These results indicate that photosynthesis and transpiration processes should be coupled in models, rather than be simulated separately, in order to precise simulation used in crop breeding. Modelling therefore enables the translation of plant biology understanding and measurement systems into decisions that improve human well-being. Modelling can also generate testable hypotheses to advance plant science, providing a blueprint for future eCO_2_ studies aimed at future-proofing crops.

## Conclusion

Confronted with the challenge of sustaining global food security, a thorough understanding of the complex interaction between maize reproductive process, eCO_2_ and drought stress is clarified. Here, we demonstrated that the potential of harnessing CO_2_ fertilization to secure reproductive success and enhance maize productivity under drought stress. eCO_2_ can enhance maize reproductive resilience to drought stress, including increasing photosynthetic efficiency and optimizing assimilate reserves in stems. These mechanisms contribute to maintaining or even increasing maize yields under drought-stressed environment, ensuring food security for a growing global population. To capitalize on the potential benefits of eCO_2_, we have discussed the importance of optimizing planting methods, mining natural genetic variation and utilizing genetic engineering techniques to develop crop varieties with improved sink strength and optimized photosynthetic systems. Additionally, we have highlighted the value of integrating advanced plant phenomics and modelling techniques in crop breeding programs, which can streamline the identification of target traits and facilitate the translation of plant biology understanding into practical applications. Ultimately, the successful adaptation of maize to elevated CO_2_ and drought stress will play a vital role in ensuring global food security in facing a rapidly changing climate.

## Author contributions

YS conceived this idea, and YL and PZ drafted the manuscript. ZZ and WS helped in drafting and collecting references. RR helped in polishing the draft. YL, PZ and YS finalized the manuscript. All authors contributed to the article and approved the submitted version.
